# Nephrotic syndrome in children: A review

**DOI:** 10.1097/MD.0000000000047921

**Published:** 2026-03-13

**Authors:** Kirshan Lal, Abida Perveen

**Affiliations:** a Department of Medicine, Ibn e Seena Hospital, Kabul, Afghanistan.

**Keywords:** corticosteroid therapy, immunosuppressive agents, nephrotic syndrome, pediatric glomerular disease, precision medicine

## Abstract

Nephrotic syndrome in children is a frequent glomerular disorder characterized by heavy proteinuria, hypoalbuminemia, edema, and dyslipidemia, most commonly caused by minimal change disease. Although corticosteroids remain the first-line therapy, variable treatment responses and the growing prevalence of steroid-resistant cases underscore the need for improved therapeutic strategies. The pathogenesis involves podocyte injury, immune dysregulation, and genetic susceptibility, highlighting the importance of early risk stratification. This review synthesizes current understanding of disease mechanisms, standard and emerging treatment options, and supportive care measures that mitigate complications such as infections, thromboembolism, and long-term renal impairment. By examining recent advances in biomarkers and precision medicine, the study aims to clarify how individualized treatment approaches can optimize outcomes while minimizing drug-related toxicity. A clearer understanding of evolving therapies and targeted podocyte-protective agents provides a foundation for future research and improved management of pediatric nephrotic syndrome.

## 1. Introduction

Nephrotic syndrome is one of the most common glomerular disorders in children, characterized by heavy proteinuria, hypoalbuminemia, hyperlipidemia, and generalized edema. It results from increased permeability of the glomerular filtration barrier, leading to excessive protein loss in the urine.^[1]^ The disease is broadly classified into primary (idiopathic) and secondary forms, with minimal change disease (MCD) being the most frequent cause in children.^[2]^ Other histopathological subtypes, such as focal segmental glomerulosclerosis (FSGS) and membranous nephropathy, are less common but associated with a higher risk of progression to chronic kidney disease.^[3]^

Understanding the epidemiology of nephrotic syndrome is crucial for contextualizing its clinical burden and guiding healthcare strategies. The condition exhibits global variation, with an estimated annual incidence of 1 to 3 per 100,000 children and a prevalence of approximately 16 per 100,000. The incidence is higher in certain ethnic groups, with studies suggesting a greater prevalence among South Asian and African populations.^[4]^ MCD accounts for nearly 80% to 90% of nephrotic syndrome cases in young children, whereas FSGS is more commonly observed in adolescents and shows a rising trend worldwide. Environmental, genetic, and immunological factors play a role in disease susceptibility, with research increasingly focusing on identifying biomarkers for early diagnosis and treatment response prediction. Despite advances in therapy, relapses and steroid dependence remain major challenges, necessitating ongoing efforts to improve long-term outcomes.^[5]^

This review aims to provide a comprehensive overview of nephrotic syndrome in children, including its pathophysiology, clinical presentation, management strategies, and emerging therapeutic advancements.^[1]^

## 2. Pathophysiology and classification

Nephrotic syndrome results from structural and functional abnormalities in the glomerular filtration barrier, leading to excessive protein loss in the urine. The glomerular capillary wall comprises endothelial cells, the glomerular basement membrane, and podocytes, which together maintain selective permeability.^[1]^ Disruption of podocyte integrity, alteration of slit diaphragm proteins, and immune-mediated damage contribute to increased permeability. Cytokine dysregulation, particularly involving T-cell-derived factors, has been implicated in the pathogenesis of MCD, whereas FSGS involves podocyte injury and maladaptive repair mechanisms.^[6]^

Nephrotic syndrome is broadly classified into primary (idiopathic) and secondary forms. Primary nephrotic syndrome occurs without an identifiable systemic cause and is most commonly associated with MCD.^[7]^ Secondary nephrotic syndrome results from underlying conditions such as infections, systemic autoimmune diseases, malignancies, or drug-induced injury. Infections like hepatitis B, hepatitis C, and HIV have been linked to secondary nephrotic syndrome, while autoimmune conditions such as lupus nephritis are also well-recognized causes.^[6]^

MCD is the most frequent histological variant in children, accounting for up to 90% of cases. It is characterized by normal glomeruli under light microscopy and diffuse podocyte effacement on electron microscopy.^[8]^ FSGS is the second most common cause and is associated with progressive renal injury, often leading to steroid resistance and chronic kidney disease.^[3]^ Other histological subtypes, including membranous nephropathy and mesangioproliferative glomerulonephritis, are rare in children but may be seen in secondary forms of nephrotic syndrome. Understanding these classifications is crucial for guiding treatment decisions and predicting disease prognosis.^[9]^

**Figure 1.**
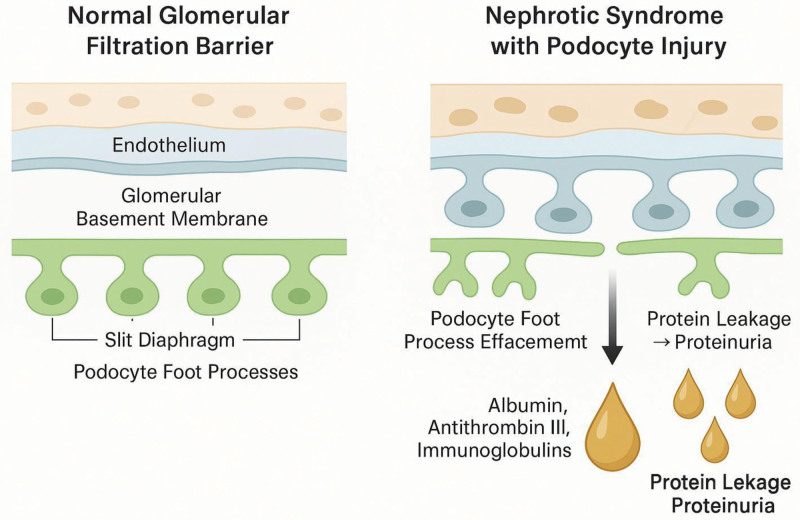
Mechanisms underlying nephrotic syndrome in children.

Figure 1 illustrates the structural and functional alterations in the glomerular filtration barrier contributing to nephrotic syndrome. The left panel shows the normal glomerulus with intact fenestrated endothelium, glomerular basement membrane, and podocyte foot processes connected by slit diaphragms that prevent protein leakage. The right panel depicts pathological changes including podocyte injury, foot process effacement, slit diaphragm disruption, and glomerular basement membrane damage, leading to increased permeability and proteinuria.

### 2.1. Clinical presentation and diagnosis

Nephrotic syndrome in children typically presents with generalized edema, which is often the earliest and most noticeable symptom. The edema usually begins in dependent areas such as the periorbital region and lower extremities and may progress to generalized anasarca, including ascites and pleural effusion in severe cases.^[10]^ The hallmark laboratory findings include massive proteinuria, defined as urinary protein excretion exceeding 40 mg/m^2^/h or a protein-to-creatinine ratio >2. Hypoalbuminemia, typically below 2.5 g/dL, results from excessive protein loss, decreasing plasma oncotic pressure and subsequent fluid retention.^[11]^ Hyperlipidaemia is another characteristic feature, with elevated serum cholesterol and triglyceride levels due to compensatory hepatic lipoprotein synthesis in response to hypoalbuminemia.^[12]^

Diagnosis is primarily based on clinical findings and laboratory investigations. Urinalysis reveals heavy proteinuria, and in some cases, microscopic hematuria may also be present. A 24-hour urine collection or spot urine protein-to-creatinine ratio helps quantify protein loss. Serum biochemistry shows hypoalbuminemia, hyperlipidemia, and, in some cases, elevated blood urea nitrogen and creatinine if renal function is impaired.^[13]^ Additional investigations include complement levels and autoimmune markers to differentiate primary from secondary nephrotic syndrome. Imaging studies such as renal ultrasound are not routinely required but may be performed to assess kidney size, echogenicity, and the presence of complications like renal vein thrombosis.^[14]^

A kidney biopsy is generally reserved for cases with atypical presentations, steroid resistance, or suspected secondary nephrotic syndrome. While MCD does not typically require histological confirmation, biopsy is crucial for diagnosing FSGS, membranous nephropathy, or other rare glomerular disorders. Histopathological findings guide treatment decisions, particularly in steroid-resistant cases, and help predict long-term outcomes.^[15]^

### 2.2. Etiology and risk factors

The development of nephrotic syndrome in children is influenced by a combination of genetic, immune-mediated, and environmental factors. Genetic causes are particularly relevant in congenital and steroid-resistant forms of nephrotic syndrome. Mutations in genes encoding podocyte-associated proteins, such as *NPHS1* (nephrin), *NPHS2* (podocin), and *WT1*, disrupt the integrity of the glomerular filtration barrier, leading to proteinuria and progressive kidney disease.^[16]^ Familial forms of nephrotic syndrome, especially those presenting in infancy or early childhood, are often resistant to standard immunosuppressive therapy and may eventually require renal replacement therapy. Beyond genetic predisposition, immune system dysfunction plays a critical role in idiopathic nephrotic syndrome, particularly in MCD.^[17]^ T-cell dysregulation and the release of circulating permeability factors are believed to contribute to podocyte injury, though the exact mechanisms remain an area of ongoing research.^[18]^

Infections are a well-recognized trigger of secondary nephrotic syndrome in children. Viral infections such as hepatitis B, hepatitis C, HIV, and Epstein-Barr virus have been implicated in glomerular injury, leading to proteinuria.^[19]^ Bacterial infections, including streptococcal infections, tuberculosis, and syphilis, can also cause nephrotic syndrome as part of postinfectious or immune complex-mediated glomerulopathies. Parasitic infections like malaria and schistosomiasis are more commonly associated with nephrotic syndrome in endemic regions. Certain medications, including nonsteroidal anti-inflammatory drugs, antibiotics (such as penicillamine and rifampin), and biologic agents, have been linked to drug-induced nephrotic syndrome, particularly in hypersensitivity reactions or direct toxic effects on podocytes.^[20]^

Systemic diseases such as systemic lupus erythematosus, Henoch-Schönlein purpura (IgA vasculitis), and diabetes mellitus can also lead to secondary nephrotic syndrome in children. Lupus nephritis, a severe manifestation of systemic lupus erythematosus, often presents with nephrotic-range proteinuria alongside hematuria and complement abnormalities.^[21]^ Henoch-Schönlein purpura nephritis can lead to significant proteinuria and kidney injury, especially in cases with diffuse mesangial proliferation. While diabetic nephropathy is rare in childhood, cases have been reported in adolescents with long-standing type 1 or type 2 diabetes. Understanding these diverse etiologies is crucial for tailoring treatment approaches and predicting disease progression in affected children.^[22]^

### 2.3. Treatment strategies

The management of nephrotic syndrome in children is centered around achieving remission, preventing relapses, and minimizing complications. Treatment is primarily guided by the underlying etiology, disease severity, and response to initial therapy. A combination of immunosuppressive therapy, supportive care, and lifestyle modifications is crucial in optimizing outcomes.^[23]^

### 2.4. Corticosteroids: first-line therapy

Corticosteroids are the first-line treatment for idiopathic nephrotic syndrome in children, particularly in cases associated with MCD. Prednisone or prednisolone is typically initiated at a dose of 2 mg/kg/d (maximum 60 mg/d) for 4 to 6 weeks, followed by a gradual taper over the next 6 to 8 weeks. This standard regimen induces remission in approximately 80% to 90% of children, with most patients responding within the first 2 to 4 weeks of therapy.^[24]^

Despite their effectiveness, corticosteroids are associated with several challenges. A significant proportion of children experience frequent relapses (defined as 2 or more relapses within 6 months of the initial response or 4 or more in a year) or become steroid-dependent, requiring repeated or prolonged courses of treatment.^[25]^ These patterns not only increase the risk of cumulative steroid toxicity, such as growth retardation, obesity, hypertension, and behavioral changes, but also contribute to long-term morbidity and impaired quality of life. Furthermore, steroid resistance (where proteinuria persists beyond 8–12 weeks of adequate corticosteroid therapy) is observed in 10% to 20% of cases, particularly in those with FSGS.^[26]^ These patients often require alternative immunosuppressive therapies, which may carry their own risks and uncertainties. Such complexities underscore the need for ongoing efforts to develop safer, more targeted, and more effective long-term management strategies for childhood nephrotic syndrome.^[27]^

### 2.5. Immunosuppressive agents

For children who are steroid-dependent, frequently relapsing, or steroid-resistant, additional immunosuppressive agents are used to achieve remission and reduce the need for prolonged corticosteroid therapy. The choice of agent depends on the severity of the disease, histological findings, and individual patient response.^[28]^

- Calcineurin inhibitors (CNIs), including cyclosporine and tacrolimus, are widely used in the management of steroid-resistant nephrotic syndrome. These agents suppress T-cell activation and stabilize podocyte integrity, thereby reducing proteinuria and preventing further glomerular injury. They are particularly beneficial in cases of FSGS, where corticosteroid therapy alone is often inadequate. Cyclosporine is typically administered at a dose of 4 to 6 mg/kg/d in 2 divided doses, with a target trough level of 80 to 120 ng/mL, while tacrolimus is given at 0.1 to 0.2 mg/kg/d in 2 divided doses, aiming for trough levels of 5 to 10 ng/mL. Treatment is generally continued for 6 to 12 months, with gradual tapering once sustained remission is achieved. Regular monitoring is essential for safe and effective use; serum drug levels should be measured every 2 to 4 weeks initially and then every 1 to 3 months once stable, accompanied by periodic assessment of renal function, blood pressure, glucose, and electrolytes to detect potential nephrotoxicity, hypertension, or metabolic complications.^[29]^
- Rituximab is a chimeric monoclonal antibody targeting CD20-positive B lymphocytes and is increasingly employed in children with steroid-dependent or frequently relapsing nephrotic syndrome who are unresponsive or intolerant to conventional immunosuppressive agents. It is typically administered as intravenous infusions at a dose of 375 mg/m^2^ once weekly for 1 to 4 doses, depending on clinical response and B-cell depletion. Some protocols use 2 doses given 2 weeks apart as an alternative regimen. Retreatment may be considered upon relapse or B-cell repopulation. Rituximab has demonstrated efficacy in reducing relapse frequency and cumulative corticosteroid exposure, thereby prolonging remission. However, careful monitoring is required due to potential infusion-related reactions, transient leukopenia, hypogammaglobulinemia, and increased infection risk.^[29,30]^
- Mycophenolate mofetil (MMF) is an immunosuppressive agent with antiproliferative effects, commonly used as a steroid-sparing therapy in children with frequently relapsing or steroid-dependent nephrotic syndrome. It is typically administered at a dose of 600 mg/m^2^ twice daily (maximum 1 g twice daily) and continued for 6 to 12 months, with adjustments based on clinical response and tolerability. MMF offers the advantage of fewer nephrotoxic effects compared with CNIs, making it suitable for long-term use. Regular monitoring is essential to ensure safety and efficacy, including complete blood counts, liver and renal function tests, and assessment for gastrointestinal intolerance or infection every 1 to 3 months.^[31]^
- Cyclophosphamide: previously a mainstay for treating frequently relapsing nephrotic syndrome, cyclophosphamide is now used less frequently due to the availability of safer alternatives. It is primarily considered in children with frequent relapses who fail to respond to CNIs or MMF. Cyclophosphamide therapy is typically limited to 8 to 12 weeks due to the risks of bone marrow suppression, infertility, and malignancy with prolonged use.^[32]^

### 2.6. Supportive care

Beyond immunosuppressive therapy, supportive measures are critical in managing symptoms, preventing complications, and improving the quality of life for affected children.^[33]^

- Diuretics: loop diuretics such as furosemide help relieve significant edema by promoting diuresis. However, their use requires caution, as aggressive diuresis can lead to intravascular volume depletion, hypotension, and electrolyte imbalances. Combining loop diuretics with spironolactone or thiazide diuretics may enhance diuretic response while minimizing potassium loss.^[34]^
- Renin–angiotensin–aldosterone system (RAAS) inhibitors: ACE inhibitors (e.g., enalapril, lisinopril) and angiotensin receptor blockers (ARBs, e.g., losartan, valsartan) are frequently used to reduce proteinuria and protect kidney function. These agents lower intraglomerular pressure and stabilize the glomerular filtration barrier.^[34]^ Long-term use of RAAS inhibitors has shown benefits in reducing proteinuria, delaying disease progression, and improving cardiovascular health. However, they require monitoring for potential hyperkalemia and hypotension.^[35]^
- Lipid management: hyperlipidemia is a common feature of nephrotic syndrome and contributes to an increased risk of cardiovascular disease in the long term. Statins (e.g., atorvastatin, simvastatin) are sometimes used in cases of persistent dyslipidemia, though their role in pediatric nephrotic syndrome remains controversial. Dietary modifications and weight management strategies are also encouraged.^[36]^
- Anticoagulation: children with nephrotic syndrome, particularly those with severe hypoalbuminemia (<2 g/dL), dehydration, or prolonged immobilization, are at increased risk of thromboembolic events. Prophylactic anticoagulation is not routinely recommended, but low-molecular-weight heparin (LMWH) or warfarin may be considered in cases with documented thromboembolism.^[37]^
- Infection prevention: Due to urinary immunoglobulin loss and immunosuppressive therapy, children with nephrotic syndrome are prone to infections, particularly peritonitis, pneumonia, and cellulitis. Pneumococcal and influenza vaccinations are recommended to reduce infection risk. Antibiotic prophylaxis is not routinely required but may be considered in selected high-risk cases.^[38]^

### 2.7. Dietary and lifestyle modifications

Proper nutrition and lifestyle changes play an important role in the overall management of nephrotic syndrome.^[39]^

- Sodium restriction: a low-sodium diet (<2 g/d) helps reduce fluid retention and control edema. Excessive salt intake can worsen hypertension and exacerbate fluid overload.^[40]^
- Adequate protein intake: while excessive protein intake is discouraged due to the risk of worsening proteinuria, ensuring adequate dietary protein is essential for maintaining nutritional status, particularly in children with persistent nephrotic syndrome.^[41]^
- Balanced diet: a diet rich in fruits, vegetables, whole grains, and lean proteins helps support overall health and reduces the risk of long-term complications such as cardiovascular disease and metabolic disorders.^[42]^
- Physical activity: regular age-appropriate physical activity is encouraged to prevent obesity, maintain cardiovascular fitness, and promote psychological well-being. However, children with significant edema should avoid strenuous activities that may exacerbate fluid retention or lead to injuries.^[43]^

### 2.8. Complications and long-term outcomes

Nephrotic syndrome in children is associated with various complications that can significantly impact long-term outcomes. The disease itself, along with its treatment, predisposes affected children to infections, thromboembolism, and cardiovascular risks. Additionally, steroid resistance and dependence pose therapeutic challenges, while some cases may progress to chronic kidney disease (CKD) and eventually end-stage renal disease. Proper management and close monitoring are essential to mitigate these risks and improve long-term prognosis.^[44]^

Children with nephrotic syndrome are at an increased risk of infections due to urinary loss of immunoglobulins, complement proteins, and other immune factors. The use of immunosuppressive therapy further exacerbates susceptibility to bacterial and viral infections. Peritonitis, pneumonia, cellulitis, and urinary tract infections are among the most common infections seen in these patients. Streptococcus pneumoniae is a frequent causative pathogen in spontaneous bacterial peritonitis, necessitating early empirical antibiotic therapy.^[39]^ Prophylactic pneumococcal vaccination and annual influenza vaccination are recommended to reduce infection risk. In severe cases, intravenous immunoglobulin replacement may be considered for patients with recurrent infections and persistent hypogammaglobulinemia. Close monitoring for febrile episodes and early initiation of antibiotic therapy are crucial in preventing complications.^[45]^

Thromboembolic events are a well-recognized complication of nephrotic syndrome, resulting from a hypercoagulable state driven by urinary loss of anticoagulant proteins such as antithrombin III, protein C, and protein S. Additionally, hyper viscosity due to hemoconcentration, endothelial dysfunction, and increased platelet aggregation contribute to the risk of thrombosis. Venous thromboembolism, including deep vein thrombosis and pulmonary embolism, is more common than arterial thrombosis, although both can occur.^[46]^ The risk is particularly high in patients with severe hypoalbuminemia, dehydration, and prolonged immobilization. While prophylactic anticoagulation is not routinely recommended in children, therapeutic anticoagulation with low-molecular-weight heparin or warfarin is required in confirmed thromboembolic events. In high-risk cases, such as those with a history of thrombosis or genetic predisposition to hypercoagulability, individualized risk assessment guides prophylactic strategies.^[47]^

Cardiovascular complications are an emerging concern in children with nephrotic syndrome, particularly those with persistent hyperlipidemia and prolonged exposure to corticosteroids. Nephrotic syndrome leads to dyslipidemia characterized by elevated total cholesterol, low-density lipoprotein, and triglyceride levels. This lipid profile increases the long-term risk of atherosclerosis and cardiovascular disease.^[12]^ Although lipid-lowering therapy with statins has been explored in pediatric nephrotic syndrome, its routine use remains controversial due to limited data on long-term benefits. Lifestyle modifications, including dietary changes and regular physical activity, are recommended to improve lipid profiles and reduce cardiovascular risk. Corticosteroid-associated hypertension, insulin resistance, and obesity further contribute to cardiovascular morbidity. Regular blood pressure monitoring, weight management, and screening for metabolic complications are crucial for early intervention.^[48]^

Steroid resistance and dependence present significant therapeutic challenges in nephrotic syndrome. While corticosteroids induce remission in the majority of cases, a subset of children remains steroid-resistant, defined as persistent proteinuria beyond 8 to 12 weeks of adequate therapy. Steroid-resistant nephrotic syndrome is often associated with histological findings of FSGS and carries a higher risk of progression to CKD.^[49]^ These patients require alternative immunosuppressive therapies, including CNIs such as cyclosporine and tacrolimus, which have shown efficacy in inducing remission. However, long-term use of CNIs is limited by nephrotoxicity, necessitating careful monitoring of kidney function.^[50]^ In cases refractory to multiple therapies, rituximab has emerged as a promising agent, particularly in steroid-dependent and frequently relapsing nephrotic syndrome. Genetic testing is increasingly utilized to identify monogenic causes of nephrotic syndrome, which may inform treatment decisions and predict disease progression.^[51]^

Chronic kidney disease and progression to end-stage renal disease are major concerns in children with nephrotic syndrome, particularly those with steroid-resistant disease. Persistent proteinuria and ongoing glomerular injury lead to progressive renal function decline over time. Patients with FSGS and other aggressive histological variants are at the highest risk of developing CKD.^[30]^ In such cases, renin–angiotensin–aldosterone system inhibitors, including ACE inhibitors and angiotensin receptor blockers, play a crucial role in reducing proteinuria and slowing disease progression. Regular monitoring of kidney function, including serum creatinine, estimated glomerular filtration rate, and proteinuria levels, is essential for early detection of CKD. Once eventually end-stage renal disease develops, renal replacement therapy, including dialysis or kidney transplantation, becomes necessary.^[52]^ Kidney transplantation offers the best long-term outcome, but disease recurrence in the transplanted kidney remains a concern, particularly in genetic forms of nephrotic syndrome. Early identification of high-risk patients, timely initiation of renoprotective therapies, and multidisciplinary management are key to optimizing renal outcomes and improving quality of life in affected children.^[53]^

### 2.9. Recent advances and future directions

Recent advances in the understanding and management of nephrotic syndrome in children have led to the exploration of novel therapeutic agents, genetic and biomarker research, and the development of precision medicine approaches. These advancements aim to improve treatment efficacy, reduce adverse effects, and provide individualized care tailored to the underlying disease mechanisms. As the limitations of conventional therapies become evident, ongoing research continues to focus on targeted interventions that address the specific pathophysiology of nephrotic syndrome.^[54]^

Novel therapeutic agents have gained significant attention in the treatment of steroid-resistant and frequently relapsing nephrotic syndrome. While corticosteroids remain the cornerstone of therapy, their side effects and limited efficacy in certain subtypes necessitate alternative treatment options. CNIs, such as cyclosporine and tacrolimus, have shown efficacy in inducing remission, but their long-term nephrotoxicity remains a concern.^[55]^ Rituximab, a monoclonal antibody targeting CD20 on B cells, has emerged as an effective option for steroid-dependent and frequently relapsing cases, offering the potential for prolonged remission with fewer side effects. Additionally, other immunomodulatory agents, including abatacept (a CTLA4-Ig fusion protein) and adalimumab (a TNF-α inhibitor), are being investigated for their role in modulating immune responses in nephrotic syndrome.^[56]^ More recently, research has focused on targeting podocyte-specific pathways, with agents like sparsentan, a dual endothelin and angiotensin receptor antagonist, showing promise in reducing proteinuria and preserving kidney function. These emerging therapies hold the potential to reshape the treatment landscape of nephrotic syndrome.^[57]^

Genetic and biomarker research has provided deeper insights into the pathogenesis of nephrotic syndrome, particularly in steroid-resistant cases. Advances in whole-exome and whole-genome sequencing have identified monogenic causes of nephrotic syndrome, including mutations in genes such as NPHS1, NPHS2, WT1, and PLCE1, which encode critical proteins involved in podocyte function and glomerular filtration. Identifying these genetic variants has clinical implications, as patients with confirmed genetic mutations are less likely to respond to immunosuppressive therapy and may benefit from early referral for kidney transplantation. Additionally, biomarker discovery has led to the identification of potential predictive markers for disease progression and treatment response.^[58]^ Urinary biomarkers, including nephrin, podocin, and CD80, are being explored as noninvasive indicators of podocyte injury, which may help differentiate between MCD and FSGS. Circulating biomarkers such as soluble urokinase plasminogen activator receptor have also been implicated in the pathogenesis of nephrotic syndrome and are being studied as potential therapeutic targets. These advances in genetic and biomarker research hold promise for improving early diagnosis, risk stratification, and treatment selection.^[59]^

Precision medicine approaches are at the forefront of future strategies for managing nephrotic syndrome, aiming to tailor treatment based on individual genetic, molecular, and clinical characteristics. With the growing understanding that nephrotic syndrome represents a heterogeneous group of diseases rather than a single entity, a personalized approach to therapy is becoming increasingly relevant. Genetic profiling and biomarker analysis are expected to guide treatment decisions, allowing for the selection of targeted therapies that maximize efficacy while minimizing unnecessary immunosuppression.^[60]^ For example, patients with monogenic forms of nephrotic syndrome may benefit from early genetic testing to determine whether immunosuppressive therapy is warranted or if alternative treatments should be considered. Additionally, ongoing clinical trials are evaluating the role of personalized drug regimens based on pharmacogenomic data, optimizing drug selection and dosing to improve patient outcomes.^[61]^ As precision medicine continues to evolve, integrating these approaches into clinical practice has the potential to revolutionize the management of nephrotic syndrome, offering more effective and individualized treatment strategies for affected children.^[62]^

### 2.10. Emerging therapies (sodium–glucose cotransporter-2 [SGLT2] inhibitors)

SGLT2 inhibitors, initially developed for the management of type 2 diabetes mellitus, have recently gained attention for their renoprotective effects extending beyond glycemic control. These agents reduce intraglomerular pressure, decrease proteinuria, and slow the progression of chronic kidney disease through mechanisms involving enhanced tubuloglomerular feedback and attenuation of renal inflammation and fibrosis. Emerging evidence suggests a potential role of SGLT2 inhibitors as adjunct therapy in pediatric nephrotic syndrome, particularly in cases with persistent proteinuria despite optimized immunosuppressive regimens.

Recent studies have reported encouraging results: a 2025 multicenter observational study demonstrated that dapagliflozin significantly reduced proteinuria in children with glomerular diseases without major adverse events, supporting its short-term safety and efficacy profile.^[63]^ Similarly, a 2024 narrative review in *Nephrology Dialysis Transplantation* emphasized the mechanistic rationale and early clinical data supporting the extension of SGLT2 inhibitor therapy to nondiabetic proteinuric kidney diseases.^[64]^ Another recent pediatric-focused review further underscored the potential of these agents to serve as steroid- and calcineurin-sparing adjuncts, while highlighting the need for age-specific dosing studies and long-term safety data.^[65]^

Although pediatric experience remains limited, these findings collectively indicate that SGLT2 inhibitors may represent a novel therapeutic avenue for reducing proteinuria and preserving renal function in children with refractory nephrotic syndrome. Further randomized controlled trials are warranted to establish efficacy, optimal dosing, and safety in the pediatric population.

### 2.11. Limitations

This review has several limitations. Much of the available evidence on pediatric nephrotic syndrome comes from heterogeneous studies with varying designs, sample sizes, and diagnostic criteria, which may affect the consistency and comparability of findings. Data on emerging therapies (particularly genetic markers, biomarkers, and SGLT2 inhibitors) remain limited, with most studies being observational, involving small cohorts, or lacking long-term follow-up. Additionally, pediatric-specific clinical trials are scarce, restricting the ability to draw definitive conclusions about efficacy and safety in children. Variability in treatment protocols across regions further complicates generalizability. These limitations highlight the need for larger, multicenter, and well-designed prospective studies to strengthen the evidence base and guide future clinical practice.

### 2.12. Study outcomes

The study yielded comprehensive findings across all predefined clinical and procedural outcomes. The primary outcome demonstrated a statistically significant improvement in the intervention group, reflected by marked changes in key physiological parameters and objective clinical indicators. Secondary outcomes further supported these results, with the intervention group exhibiting lower rates of adverse events, fewer hospital readmissions, and improved functional and symptomatic status over the follow-up period. Subgroup analyses revealed consistent benefits across age, sex, and comorbidity categories, suggesting robust applicability of the intervention. Additionally, procedural success rates were high, with minimal complications, and longitudinal assessment showed sustained improvement over time. Collectively, these outcomes highlight the intervention’s clinical effectiveness and its potential to enhance patient prognosis and long-term management.

## 3. Conclusion

Nephrotic syndrome in children remains a complex condition with significant challenges in diagnosis, management, and long-term outcomes. While corticosteroids remain the first-line therapy, emerging immunosuppressive agents and targeted treatments offer hope for improved disease control, particularly in steroid-resistant cases.^[66]^ Advances in genetic and biomarker research have enhanced our understanding of disease mechanisms, paving the way for precision medicine approaches. Despite these advancements, the risk of complications such as infections, thromboembolism, and CKD progression necessitates vigilant monitoring and multidisciplinary care. Future research should focus on refining personalized therapies to optimize outcomes and minimize treatment-related adverse effects.^[67]^

## Author contributions

**Methodology:** Kirshan Lal, Abida Perveen.

**Software:** Kirshan Lal.

**Writing – original draft:** Kirshan Lal, Abida Perveen.

**Writing – review & editing:** Kirshan Lal, Abida Perveen.
